# Early Prediction of Preeclampsia

**DOI:** 10.1155/2014/297397

**Published:** 2014-07-17

**Authors:** Leona C. Poon, Kypros H. Nicolaides

**Affiliations:** Harris Birthright Research Centre of Fetal Medicine, King's College Hospital, Denmark Hill, London SE5 9RS, UK

## Abstract

Effective screening for the development of early onset preeclampsia (PE) can be provided in the first-trimester of pregnancy. Screening by a combination of maternal risk factors, uterine artery Doppler, mean arterial pressure, maternal serum pregnancy-associated plasma protein-A, and placental growth factor can identify about 95% of cases of early onset PE for a false-positive rate of 10%.

## 1. Introduction

Preeclampsia (PE) is a major cause of maternal and perinatal morbidity and mortality [1–3] and is thought to be predominantly as the consequence of impaired placentation. Evidence suggests that PE can be subdivided into early onset PE, requiring delivery before 34 weeks' gestation and late onset PE, with delivery at or after 34 weeks, because the former is associated with a higher incidence of adverse outcome [4–7]. A major challenge in modern obstetrics is early identification of pregnancies at high-risk of early onset PE and undertaking the necessary measures to improve placentation and reduce the prevalence of the disease.

The prophylactic use of low-dose aspirin for prevention of PE has been an important research question in obstetrics for the last three decades. In 1979, Crandon and Isherwood observed that nulliparous women who had taken aspirin regularly during pregnancy were less likely to have PE than women who did not. Subsequently, more than 50 trials have been carried out throughout the world and a meta-analysis of these studies reported that the administration of low-dose aspirin in high-risk pregnancies is associated with a decrease in the rate of PE by approximately 10% [8]. In most studies that evaluated aspirin for the prevention of PE the onset of treatment was after 16 weeks' gestation. However, recent meta-analyses reported that the prevalence of PE can potentially be halved by the administration of low-dose aspirin started at 16 weeks or earlier [9–11].

Extensive research in the last 20 years, mainly as a consequence of the shift in screening for aneuploidies from the second- to the first-trimester of pregnancy, has identified a series of early biophysical and biochemical markers of impaired placentation [12, 13]. Using a novel Bayes-based method that combines prior information from maternal characteristics and medical history, uterine artery pulsatility index (PI), mean arterial pressure (MAP), and maternal serum pregnancy-associated plasma protein-A (PAPP-A) and placental growth factor (PlGF) at 11–13 weeks' gestation can identify a high proportion of pregnancies at high-risk for early onset PE [12, 13]. The performance of the different methods of screening for PE is summarized in Table 1.

## 2. Screening by Maternal History

Several professional bodies have issued guidelines on routine antenatal care recommending that, at the booking visit, a woman's level of risk for PE, based on factors in her history, should be determined and women at high-risk are advised to take low-dose aspirin daily from early pregnancy until the birth of the baby (Table 2) [14–17]. However, the performance of screening by the recommended method and the effectiveness of intervention have not been formally evaluated.

The majority of the studies that have reported on the maternal risk factors for the development of PE do not quantify the risk, although some studies do provide relative risks. Most of the available literature is based on retrospective, epidemiological, cohort, or case-control studies though few prospective cohort studies are also reported. Only a few studies have reported on maternal risk factors according to the severity of the disease, that is, early onset PE versus late onset PE.

**Figure 1:**
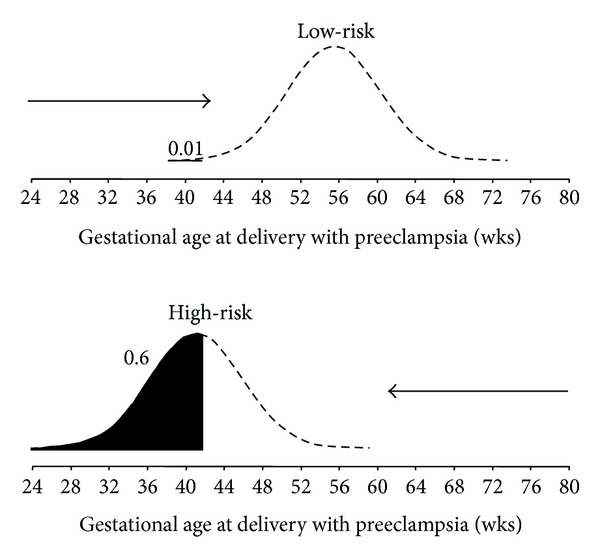
Distribution of gestational age at delivery for preeclampsia (PE). In pregnancies at low-risk for PE the gestational age distribution is shifted to the right and in most pregnancies delivery will occur before the development of PE. In pregnancies at high-risk for PE the distribution is shifted to the left. The risk of PE occurring at or before a specified gestational age is given by the area under the distribution curve (black). In the low-risk group the risk of PE at or before 34 weeks' gestation is 0.01 or 1% and in the high-risk group the risk is 0.6 or 60%.

It has been demonstrated that maternal demographic characteristics, including medical and obstetric history (Table 2), are potentially useful in screening for PE only when the various factors are incorporated into a combined algorithm derived by multivariate analysis [18]. With this approach to screening the effects of variables are expressed as odds ratios for early onset, late onset, or total PE. In general, the maternal risk factor profiles vary between early onset PE and late onset PE. This has led to the view that early and late PE may be different diseases. An alternative view is that PE is a spectrum disorder the degree of which is reflected in gestational age at the time of delivery. Multivariate screening for PE with maternal risk factors has since evolved into a new approach in which the gestation at the time of delivery for PE is treated as a continuous rather than a categorical variable. This approach, which is based on a survival time model, assumes that if the pregnancy was to continue indefinitely, all women would develop PE and whether they do so or not before a specified gestational age depends on a competition between delivery before or after development of PE [12]. In this new approach the effect of various risk factors is to modify the mean of the distribution of gestational age at delivery with PE. In pregnancies at low-risk for PE the gestational age distribution is shifted to the right with the implication that in most pregnancies delivery will actually occur before the development of PE. In high-risk pregnancies the distribution is shifted to the left and the smaller the mean gestational age, the higher the risk for PE (Figure 1).

**Figure 2:**
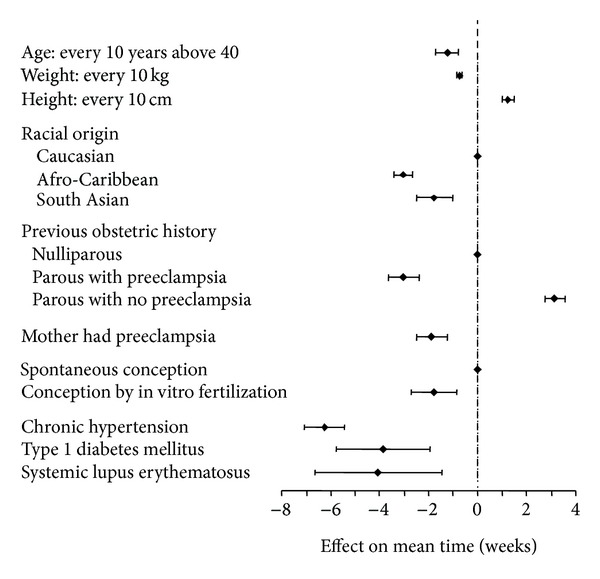
Effects of maternal characteristics (with 95% confidence intervals) on the gestational age at delivery for preeclampsia. This effect is expressed as gestational weeks by which the expected gestational age at delivery for preeclampsia is altered.

In this competing risk model the mean gestational age for delivery with PE is 54 weeks with estimated standard deviation of 6.9 weeks. Certain variables, including advancing maternal age over 35 years, increasing weight, Afro-Caribbean and South Asian racial origin, previous pregnancy with PE, conception by in vitro fertilization (IVF) and a medical history of chronic hypertension, preexisting diabetes mellitus, and systemic lupus erythematosus or antiphospholipid syndrome, increase the risk for development of PE. The consequence of this increased risk is a shift to the left of the Gaussian distribution of the gestational age at delivery with PE (Figure 2). Estimated detection rates of PE requiring delivery before 34, 37, and 42 weeks' gestation in screening by maternal factors are about 36%, 33%, and 29%, respectively, at false-positive rate of 5%, and 51%, 43%, and 40%, respectively, at false-positive rate of 10% (Table 1) [12].

## 3. Screening by Maternal Biophysical Markers

### 3.1. Uterine Artery Doppler

The most promising screening test for PE is uterine artery Doppler velocimetry. The spiral arteries undergo a series of morphological changes during normal pregnancy [19, 20]. The vessels are firstly invaded by trophoblast, which then becomes incorporated into the vessel wall and replaces the endothelium and muscular layer. This results in the conversion of the small spiral arteries into vessels of greater diameter with low resistance and high compliance, in absence of maternal vasomotor control. This vascular transformation in the uterus is necessary to ensure a dramatic increase in blood supply to the intervillous space. The underlying mechanism for the development of PE is thought to be impaired trophoblastic invasion of the maternal spiral arteries and their conversion from narrow muscular vessels to wide nonmuscular channels [21–25]. Doppler ultrasound provides a noninvasive method for the assessment of the uteroplacental circulation. The finding that impaired placental perfusion, reflected in increased uterine artery PI, is associated with the development of PE is compatible with the hypothesis that PE is the consequence of impaired placentation and the results of previous first- and second-trimester Doppler studies as well as histological studies of the maternal spiral arteries [26–29]. Pathological studies have demonstrated that the prevalence of placental lesions in women with PE is inversely related to the gestation at delivery [30, 31].

The ability to achieve a reliable measurement of uterine artery PI is dependent on appropriate training of sonographers, adherence to a standard ultrasound technique in order to achieve uniformity of results among different operators. Using transabdominal ultrasonography, a sagittal section of the uterus should be obtained and the cervical canal and internal cervical os are identified. Subsequently, the transducer is gently tilted from side to side and color flow mapping is used to identify each uterine artery along the side of the cervix and uterus at the level of the internal os. Pulsed wave Doppler is then used with the sampling gate set at 2 mm to cover the whole vessel and care should be taken to ensure that the angle of insonation is less than 30°. When three similar consecutive waveforms are obtained the PI is measured and the mean PI of the left and right arteries is calculated. It is important to ensure that the peak systolic velocity is greater than 60 cm/s to ensure that the arcuate artery is not being sampled instead of the uterine artery [29].

First-trimester uterine artery PI has been shown to be affected by gestational age at screening, maternal weight, racial origin, and history of preexisting diabetes mellitus, and consequently it should be expressed as multiple of median (MoM) after adjustment for these factors. The MoM value of uterine artery PI is significantly increased at 11–13 weeks' gestation in women who subsequently develop PE and there is a significant negative linear correlation between the uterine artery PI MoM with gestational age at delivery [12]. Estimated detection rates of PE, at false-positive rate of 5% and 10% in screening by maternal factors with uterine artery PI, are given in Table 1. The addition of uterine artery PI to maternal factors improves the detection rates from 36% to 59% and from 33% to 40%, at false-positive rate of 5%, and from 51% to 75% and from 43% to 55%, at false-positive rate of 10%, for PE requiring delivery before 34 and 37 weeks' gestation, respectively, but not for PE delivering before 42 weeks.

### 3.2. Blood Pressure

In PE, hypertension develops as a result of vasoconstriction and reduced peripheral vascular compliance [32]. Although hypertension is only a secondary sign of PE, it is an important sign as it is an early indication of the disease. This highlights the importance of accurate monitoring of blood pressure during antenatal care. Accurate assessment of blood pressure has been hindered by the considerable variability that blood pressure exhibits within each individual. During blood pressure measurement at rest the first recording is often the highest recording, which decreases as the patients become more familiar with the procedure [33]. It is therefore recommended by professional bodies that a series of blood pressure measurements should be made until a prespecified level of stability is achieved [34, 35]. In current clinical practice, the use of mercury sphygmomanometers remains the gold standard for noninvasive blood pressure monitoring, but there are concerns for both the clinical performance and safety of these instruments [36–38]. Observer error is a major limitation of the auscultatory method [39] and terminal digit preference is perhaps the most common manifestation of suboptimal blood pressure determination. Other considerations include the rate of cuff deflation, the use of correct size cuff, the interarm difference in blood pressure, and the arm position and posture that are recognized to have significant effects on blood pressure determination.

The introduction of automated blood pressure monitoring allows simple, standardized, and repeated measurements to be taken. It also addresses many of the errors associated with the conventional sphygmomanometer but their use still requires the selection of the correct cuff size and proper patient positioning if accurate blood pressure is to be obtained. It has therefore been proposed that MAP should be measured by validated automated devices [40], with women in sitting position with back supported and legs uncrossed, that two measurements should be taken from each arm simultaneously with each arm supported at the level of the heart, and that the average of the four measurements should be used [33].

There is substantial evidence demonstrating that an increase in blood pressure in women destined to develop PE can be observed in the first- and second-trimesters of pregnancy [41–75]. Previous studies, including a mixture of prospective and retrospective and cohort and case-control studies and randomized controlled trials, reported widely contradictory results in the performance of screening (detection rate, median 43%, range 5–100%; false-positive rate, median 16%, range 0–66%) as a consequence of major methodological differences. The data from these studies, including more than 60,000 women with 3,300 cases of PE, were compiled into a systematic review, which concluded that the MAP is significantly better than systolic blood pressure or diastolic blood pressure in predicting PE [76].

First-trimester MAP has been shown to be affected by maternal weight, height, age, racial origin, cigarette smoking, family and prior history of PE, and history of chronic hypertension, and consequently it should be expressed as MoM after adjustment for these factors. Similar to the findings with uterine artery PI, the MoM value of MAP is significantly increased at 11–13 weeks' gestation in women who subsequently develop PE and there is a significant negative linear correlation between the MAP MoM with gestational age at delivery [12]. Estimated detection rates of PE, at false-positive rate of 5% and 10% in screening by maternal factors with MAP, are given in Table 1. The addition of MAP to maternal factors improves the detection rates from 36% to 58%, from 33% to 44%, and from 29% to 37%, at false-positive rate of 5%, and from 51% to 73%, from 43% to 59%, and from 40% to 54%, at false-positive rate of 10%, for PE requiring delivery before 34, 37, and 42 weeks' gestation, respectively.

There is a significant association between uterine artery PI and MAP in PE and unaffected pregnancies and therefore when combining the two biophysical markers in calculating the patient specific risk for PE the correlation factors must be taken into consideration to avoid overestimating the contributions from each marker in order to provide accurate risk assessment for PE. Estimated detection rates of PE requiring delivery before 34, 37, and 42 weeks' gestation in screening by maternal factors are 80%, 55%, and 35%, respectively, at false-positive rate of 5% and 90%, 72%, and 57%, respectively, at false-positive rate of 10% (Table 1).

## 4. Screening by Maternal Biochemical Markers

A large number of biochemical markers have been investigated for the prediction of PE (Table 3). Many such markers represent measurable manifestations of impaired placentation due to inadequate trophoblastic invasion of the maternal spiral arteries and reduced placental perfusion leading to placental ischemia related damage with the release of inflammatory factors, platelet activation, endothelial dysfunction, maternal renal dysfunction, or abnormal oxidative stress [19, 21–25]. Maternal serum PAPP-A and PlGF are two biochemical markers that have been investigated extensively and have shown promising results in the early prediction of PE. They have both been shown to be useful in screening for aneuploidies in combination with maternal age, fetal nuchal translucency thickness, and maternal serum free *β*-human chorionic gonadotropin at 11–13 weeks' gestation [77] and they are now part of the platform of automated machines that provide reproducible results within 30–40 minutes of sampling.

PAPP-A is a syncytiotrophoblast-derived metalloproteinase, which enhances the mitogenic function of the insulin-like growth factors by cleaving the complex formed between such growth factors and their binding proteins [78, 79]. The insulin-like growth factor system is believed to play an important role in placental growth and development; it is therefore not surprising that low serum PAPP-A is associated with a higher incidence of PE. Increased level of maternal serum PAPP-A has been observed in established PE [80–82]. In chromosomally normal pregnancies there is evidence that low maternal serum PAPP-A in the first- and second-trimesters is associated with increased risk for subsequent development of PE. However, measurement of PAPP-A alone is not an effective method of screening for PE because only 8–23% of affected cases have serum levels below the 5th percentile, which is about 0.4 MoM. At the 5th percentile of normal for PAPP-A the reported odds ratios for PE varied between 1.5 and 4.6 [83–89].

PlGF, a glycosylated dimeric glycoprotein, is a member of the vascular endothelial growth factor subfamily. It binds to vascular endothelial growth factor receptor-1 which has been shown to rise in pregnancy. PlGF is synthesized in villous and extravillous cytotrophoblast and has both vasculogenetic and angiogenetic functions. It is believed to contribute a change in angiogenesis from a branching to a nonbranching phenotype controlling the expansion of the capillary network. Its angiogenetic abilities have been speculated to play a role in normal pregnancy and changes in the levels of PlGF or its inhibitory receptor have been implicated in the development of PE [90–93]. PE is associated with reduced placental production of PlGF and several studies reported that during the clinical phase of PE the maternal serum PlGF concentration is reduced. These reduced levels of serum PlGF precede the clinical onset of the disease and are evident from both the first- and second-trimesters of pregnancy [94–102].

In biochemical testing it is necessary to make adjustments in the measured maternal serum metabolite concentration to correct for certain maternal and pregnancy characteristics as well as the machine and reagents used for the assays and is then expressed in MoM of the normal [103]. First-trimester maternal serum concentrations of PAPP-A and PlGF have been shown to be affected by gestational age at screening, maternal weight, racial origin, cigarette smoking, conception by IVF, nulliparity, and preexisting diabetes mellitus [103, 104]. In addition, serum PlGF is also affected by maternal age [104]. Consequently, the measured concentrations of PAPP-A and PlGF must be adjusted for these variables before comparing results with pathological pregnancies. Contrary to the findings with biophysical markers, the MoM values of PAPP-A and PlGF are significantly reduced at 11–13 weeks' gestation in women who subsequently develop PE. There is a significant positive linear correlation between the MoM values of these biochemical markers with gestational age at delivery [13]. This observation further confirms that PE is a single pathophysiological entity with a wide spectrum of severity manifested in gestational age at which delivery becomes necessary for maternal and/or fetal indications.

Estimated detection rates of PE, at false-positive rate of 5% and 10% in screening by maternal factors with biochemical markers, are given in Table 1. The addition of maternal serum PAPP-A and PlGF to maternal factors improves the detection rates from 36% to 60% and from 33% to 43%, at false-positive rate of 5%, and from 51% to 74% and from 43% to 56%, at false-positive rate of 10%, for PE requiring delivery before 34 and 37 weeks' gestation, respectively, but not for PE delivering before 42 weeks.

## 5. Screening by Maternal Biochemical and Biophysical Markers

Analogous to the effective first-trimester combined screening for aneuploidies, effective screening for PE can also be achieved by a combination of maternal factors and biochemical and biophysical markers. Using the competing risk model, the gestational age at the time of delivery for PE is treated as a continuous variable. Bayes theorem is then used to combine prior information from maternal characteristics and medical history with the MoM values of uterine artery PI, MAP, serum PAPP-A, and PlGF. The major advantage of this model, compared to the other published models [105–107], is that it offers the option to clinicians and researchers to select their own gestational age cut-off to define the high-risk group that could potentially benefit from therapeutic interventions starting from the first-trimester of pregnancy [9–11].

It is important to recognize that there are significant associations between all biophysical and biochemical markers in PE and unaffected pregnancies and therefore when combining the four markers in calculating the patient specific risk for PE the correlation factors are taken into account to provide accurate risk assessment for PE. Estimated detection rates of PE requiring delivery before 34, 37, and 42 weeks' gestation in screening by maternal factors are 93%, 61%, and 38%, respectively, at false-positive rate of 5% and 96%, 77%, and 54%, respectively, at false-positive rate of 10% (Table 1).

## 6. First-Trimester Screening Followed by Third-Trimester Risk Assessment

Effective screening for early onset PE can be achieved in the first-trimester of pregnancy but late onset PE requiring delivery after 34 weeks' gestation accounting for two-thirds of all PE remains a significant challenge for effective early screening. We have therefore proposed a two-stage strategy for identification of pregnancies at risk of PE. The first stage, at 11–13 weeks, should be primarily aimed at effective prediction of early onset PE, because the prevalence of this condition can be potentially reduced substantially by the prophylactic use of low-dose aspirin started before 16 weeks' gestation [9–11]. The second stage, at 30–33 weeks, should be aimed at effective prediction of PE requiring delivery at or after 34 weeks because close monitoring of such pregnancies for earlier diagnosis of the clinical signs of the disease could potentially improve perinatal outcome through such interventions as the administration of antihypertensive medication and early delivery [108].

A competing risk model, using Bayes theorem, has been developed that combines maternal characteristics and history with biophysical and biochemical markers at 30–33 weeks' gestation to estimate the risk of developing PE requiring delivery within selected intervals from the time of screening. Preliminary results to date confirm that the* a priori* risk for PE depends on maternal characteristics and is increased with increasing maternal age and weight and in women of Afro-Caribbean and South Asian racial origin, in those with personal or family history of PE, and in women with preexisting chronic hypertension, diabetes mellitus, and systemic lupus erythematosus or antiphospholipid syndrome [109]. The third-trimester uterine artery PI and MAP are affected by maternal characteristics and history and the corrected measurements as expressed in MoM values are inversely related to the severity of the disease reflected in the gestational age at delivery. At risk cut-off of 1 : 100, the estimated false-positive and detection rates for PE requiring delivery within the subsequent four weeks were 6% and 91% in screening by a combination of maternal factors, uterine artery PI, and MAP [109].

PE is thought to be the consequence of an imbalance in angiogenic and antiangiogenic proteins [110]. Recent studies have focused on the investigation of pregnancies presenting to specialist clinics with signs of hypertensive disorders with the aim of identifying the subgroup that will develop severe PE requiring delivery within the subsequent 1–4 weeks. In such high-risk pregnancies, measurement of serum PlGF or soluble fms-like tyrosine kinase-1 (sFlt-1) to PlGF ratio is highly accurate in identifying the target group [111–116]. We have demonstrated that serum PlGF decreases with gestational age and maternal weight and is higher in women of Afro-Caribbean and South Asian racial origin than in Caucasians, in parous than nulliparous women, and in smokers than in nonsmokers. Serum sFlt-1 increases with gestational age and maternal age, decreases with maternal weight, is increased in women of Afro-Caribbean racial origin and in pregnancies conceived by IVF, and is lower in parous than nulliparous women [117]. In pregnancies complicated by PE, compared to normal pregnancies, serum PlGF MoM is decreased and sFlt-1 MoM is increased. At risk cut-off of 1 : 100, the estimated false-positive and detection rates for PE requiring delivery within the subsequent four weeks were 4% and 93% in screening by maternal factors, serum PlGF, and sFlt-1 [83] and the false-positive and detection rates improved to 2% and 95% in screening by maternal factors with all biomarkers [118].

## 7. Conclusion

Effective screening for early onset PE can be achieved in the first-trimester of pregnancy with a detection rate of about 95% and a false-positive rate of 10%. In a proposed new approach to prenatal care the potential value of an integrated clinic at 11–13 weeks' gestation in which maternal characteristics and history are combined with the results of a series of biophysical and biochemical markers to assess the risk for a wide range of pregnancy complications has been extensively documented [119]. In the context of PE the primary aim of such clinic is to identify those cases that would potentially benefit from prophylactic pharmacological interventions to improve placentation; the value of early screening and treatment of the high-risk group with low-dose aspirin is the subject of an ongoing randomized multicentre European study.

It is likely that a similar integrated clinic at 30–33 weeks will emerge for effective prediction of pregnancy complications that develop during the third-trimester. The potential value of such a clinic is to improve perinatal outcome by rationalizing and individualizing the timing and content of subsequent visits for selection of the best time for delivery.

## Figures and Tables

**Table 1:** Estimated detection rates of preeclampsia (PE) requiring delivery before 34, 37, and 42 weeks' gestation, at false positive rates (FPR) of 5% and 10%.

Screening test	FPR (%)	Detection rate (%)		
		PE < 34 weeks	PE < 37 weeks	PE < 42 weeks
Maternal characteristics	5.0	36	33	29
	10.0	51	43	40
Uterine artery pulsatility index (Ut-PI)	5.0	59	40	31
	10.0	75	55	42
Mean arterial pressure (MAP)	5.0	58	44	37
	10.0	73	59	54
Pregnancy associated plasma protein-A (PAPP-A)	5.0	44	37	32
	10.0	55	48	42
Placental growth factor (PlGF)	5.0	59	41	29
	10.0	72	54	40
MAP and Ut-PI	5.0	80	55	35
	10.0	90	72	57
PAPP-A and PlGF	5.0	60	43	30
	10.0	74	56	41
Ut-PI, MAP, and PAPP-A	5.0	82	53	36
	10.0	93	75	60
Ut-PI, MAP, and PlGF	5.0	87	61	38
	10.0	96	77	53
Ut-PI, MAP, PAPP-A, and PlGF	5.0	93	61	38
	10.0	96	77	54

**Table 2:** Recognized maternal risk factors for preeclampsia [14–17].

(i) Previous preeclampsia (PE)	
(ii) Previous early onset PE and preterm delivery at <34 weeks' gestation	
(iii) PE in more than one prior pregnancy	
(iv) Chronic kidney disease	
(v) Autoimmune disease such as systemic lupus erythematosis or antiphospholipid syndrome	
(vi) Heritable thrombophilias	
(vii) Type 1 or type 2 diabetes	
(viii) Chronic hypertension	
(ix) First pregnancy	
(x) Pregnancy interval of more than 10 years	
(xi) New partner	
(xii) Reproductive technologies	
(xiii) Family history of PE (mother or sister)	
(xiv) Excessive weight gain in pregnancy	
(xv) Infection during pregnancy	
(xvi) Gestational trophoblastic disease	
(xvii) Multiple pregnancies	
(xviii) Age 40 years or older	
(xix) Ethnicity: Nordic, Black, South Asian, or Pacific Island	
(xx) Body mass index of 35 kg/m^2^ or more at first visit	
(xxi) Booking systolic blood pressure >130 mmHg or diastolic blood pressure >80 mmHg	
(xxii) Increased prepregnancy triglycerides	
(xxiii) Family history of early onset cardiovascular disease	
(xxiv) Lower socioeconomic status	
(xxv) Cocaine and methamphetamine use	
(xxvi) Nonsmoking	

**Table 3:** Proposed maternal biochemical markers for the prediction of preeclampsia.

A disintegrin and metalloprotease 12 (ADAM12)	L-Arginine
Activin-A	L-Homoarginine
Adiponectin	Leptin
Adrenomedullin	Magnesium
Alpha fetoprotein	Matrix metalloproteinase-9
Alpha-1-microglobulin	Microalbuminuria
Ang-2 angiopoietin-2	Microtransferrinuria
Antiphospholipid antibodies	N-Acetyl-*β*-glucosaminidase
Antithrombin III	Neurokinin B
Atrial natriuretic peptide	Neuropeptide Y
Beta2-microglobulin	Neutrophil gelatinase-associated lipocalin
C-reactive protein	P-Selectin
Calcium	Pentraxin 3
Cellular adhesion molecules	**Placenta growth factor**
Circulating trophoblast	Placental protein 13
Corticotropin release hormone	Plasminogen activator inhibitor-2
Cytokines	Platelet activation
Dimethylarginine (ADMA)	Platelet count
Endothelin	**Pregnancy associated plasma ** **protein-A**
Estriol	Prostacyclin
Ferritin	Relaxin
Fetal DNA	Resistin
Fetal RNA	Serum lipids
Free fetal hemoglobin	Soluble endoglin
Fibronectin	Soluble fms-like tyrosine kinase
Genetic markers	Thromboxane
Haptoglobin	Thyroid function
Hematocrit	Total proteins
Homocysteine	Transferrin
Human chorionic gonadotropin	Tumor necrosis factor receptor-1
Human placental growth hormone	Uric acid
Inhibin A	Urinary calcium to creatinine ratio
Insulin-like growth factor	Urinary kallikrein
Insulin-like growth factor binding protein	Vascular endothelial growth factor
Insulin resistance	Visfatin
Isoprostanes	Vitamin D
